# Effects of various logistics packaging on the quality and microbial variation of bigeye tuna (*Thunnus obesus*)

**DOI:** 10.3389/fnut.2022.998377

**Published:** 2022-09-09

**Authors:** Zhizhen Cheng, Weicong Pan, Wendong Xian, Jin Yu, Xudong Weng, Soottawat Benjakul, Alessandra Guidi, Xiaoguo Ying, Shanggui Deng

**Affiliations:** ^1^Zhejiang Provincial Key Laboratory of Health Risk Factors for Seafood, Collaborative Innovation Center of Seafood Deep Processing, College of Food and Pharmacy, Zhejiang Ocean University, Zhoushan, China; ^2^Longyou Aquaculture Development Center, Agricultural and Rural Bureau of Longyou County, Quzhou, China; ^3^International Center of Excellence in Seafood Science and Innovation, Faculty of Agro-Industry, Prince of Songkla University, Hat Yai, Songkhla, Thailand; ^4^Department of Agriculture, Food and Environment (DAFE), Pisa University, Pisa, Italy

**Keywords:** bigeye tuna (*Thunnus obesus*), packaging materials, aluminum foil paper, quality, microbial diversity

## Abstract

Bigeye tuna (*Thunnus obesus*) is an economically valuable ocean fish species. It is susceptible to contamination during storage and transportation. Having proper transportation packaging and stable temperature during transportation are critical to prevent quality deterioration. However, the influence of packaging on retaining freshness in transit remains unknown. Here, the impact of different transportation packaging on the quality and microbiological variation of bigeye tuna during the logistics process was investigated by measuring physical-chemical indexes and microbial diversity. It turned out that aluminum foil paper (AFP) group had minimum temperature fluctuation, exhibited preferable water retaining capacity and color protection effect. AFP packaging could efficiently prevent TVB-N increase and microbial growth. After 40 h, the TVB-N value was 21.28 mg/100 g and microbial total plate count was 3.53 lg CFU/g, which was within the acceptable range. Temperature fluctuations and packaging materials had a major effect on the microbial community structure of bigeye tuna. *Chitinophagaceae, Acinetobacter*, and *Knoellia* were dominant in the AFP group, while *Pseudomonas, Acinetobacter*, and *Macrococcus* were dominant in the expanded polystyrene foam (EPSF) and European logistics (EUL) groups. AFP packaging could effectively slow down the growth and reproduction of *Pseudomonas*, restraining the growth of microorganisms and preserve the quality of bigeye tuna. This study provides insights into understanding the effects of packaging material on maintaining quality during logistics transportation.

## Introduction

Bigeye tuna (*Thunnus obesus*) is among the world's major catching fishes listed by the Food and Agriculture Organization of the United Nations (FAO). They are popular with consumers owing to their high content of n-3 polyunsaturated fatty acids, such as docosahexaenoic acid (DHA) and eicosapentaenoic acid (EPA), which are in high demand (1). According to a report by “Fishery and aquaculture statistics 2019,” the global bigeye tuna catch was 424,644 t in 2018 and 391,953 t in 2019 (2). In 2020, the catch was 30.62% lower than that in 2019, meaning that its resources were decreasing. Therefore, rational resource management is imminent. However, tuna meat is prone to oxidation, discoloration, and contamination. The growth and metabolism of microorganisms can easily be impacted by environmental changes. For example, temperature fluctuations during transit can cause stench and spoilage (3). According to Bai et al. (4), nano packaging materials can effectively maintain the quality of agricultural products during distribution. The lower the phase change temperature of refrigerated materials, the longer will the quality of agricultural products be maintained. Packaging with better insulation materials can prolong the shelf life of fish meat. The quality of aquatic products is greatly affected by temperature (5). Wang et al. (6) concluded that the temperature and its fluctuation frequency accelerated the quality deterioration of tuna. Temperature fluctuation should be as much as possible avoided to maintain the freshness of bigeye tuna in the cold chain process. As a necessary condition for transportation, the cold chain logistics box can maintain a constant temperature during transportation and thus safeguard the quality of bigeye tuna.

On the market, the most commonly used cold chain logistics boxes are European logistics (EUL) boxes, expanded polystyrene foam (EPSF) boxes, and aluminum foil paper (AFP) boxes. EUL boxes are made entirely of high-density polyethylene, which has excellent chemical resistance. In conventional seafood e-commerce supply, merchants generally adopt the distribution method of adding ice bags in the logistics box. However, when goods are delivered to consumers, ice bags would sometimes have melted. EPSF boxes have been widely used in the transportation of various aquatic and agricultural products, as well as other long-distance preservation and cold chain transportation applications. It is the most frequently used packaging box for the transportation of fresh aquatic items, as it provides thermal insulation, seismic protection, and moisture resistance. Compared to materials like plastic foam, aluminum foil is environment friendly, pollution-free, and lightweight. It can be used with other materials to increase transportation and energy efficiency, which is consistent with the prevalent notions of green consumption and sustainable development. As a result of the COVID-19 outbreak, food packaging safety is now a major concern. AFP can protect products from contamination and physical damage, withstand gas and water vapor transmission (7). AFP boxes made with new high-density synthetic material are no radiation, odor, or pollution, and exhibit high strength compression and good shock mitigation effect. AFP boxes have an excellent cooling effect, which makes it easy to transport and maintain the freshness of food. AFP boxes prolong the preservation time, delay the growth and reproduction of microorganisms to a certain degree, preserve fresh aquatic products, and prolong their shelf life.

Microorganisms play an essential role in food spoilage. The next-generation sequencing technology improved our understanding of the community-wide microbial diversity. It has been proved to be more efficient than conventional methods for analyzing the dynamics of bacterial communities in meat (8). Yu et al. found that chitooligosaccharides coating combined with ultrasound treatment of grass carp inhibited *Aeromonas* and *Shewanella* growth in refrigerated carp filets (9). Ekonomou et al. concluded that bacterial counts related to de Man-Rogosa-Sharpe (MRS) agar were the dominant methods of cultivable microbiota in chill-stored vacuum-packed hot-smoked rainbow trout filets (10). Jaaskelainen et al. claimed that the dominating bacteria in tuna were *Pseudomonas* (11). Presently, the microbial diversity analysis of tuna focused only on the cold storage stage. However, the microorganism dynamics of different packaging materials during circulation are yet to be studied. Using high-throughput sequencing technology to study the changes of flora during the circulation of aquatic products packaged in different materials is a novel perspective for understanding food preservation.

Few studies have focused on the buffering effect of different transportation packaging materials on the deterioration of bigeye tuna quality in the transportation process. Therefore, in this study, we compared the effects of different boxes on the quality and microbiological variation of bigeye tuna. We used high-throughput sequencing of 16S rRNA genes to explore the changes of microorganisms in tuna in different packaging materials during circulation. additionally, the quality change of bigeye tuna was comprehensively analyzed by real-time monitoring of the temperature changes in the packaging box and measuring numerous physical and chemical indicators such as TVB-N, water holding capacity (WHC), metmyoglobin, myofibrillar protein content, microbial total plate count, and the electronic nose (E-nose). We aimed to provide a reference for the best transportation of bigeye tuna. The impact of overall quality and microbial variations of bigeye tuna revealed the microbial spoilage and quality change under various transportation packaging conditions. It provided a theoretical foundation for different packaging materials to maintain bigeye tuna transportation quality.

## Materials and methods

### Materials, packaging, and experimental design

Bigeye tuna (*Thunnus obesus*) were purchased from the Guangdong Shunxin Sea Fishery Group Co., Ltd. (Guangdong province, China), picked tuna filets with bright color and no muscle congestion and put them in polyethylene sealing bags, weighed ~500 g per bag. All samples were packed in perforated polystyrene boxes with dry ice and shipped to the laboratory of Zhejiang Ocean University. The samples were stored at −55°C in the laboratory until used.

EUL boxes (outer dimension: 300×200×120 mm, inner dimension: 250×145×125 mm) were purchased from Yancheng Tengle Plastic Industry Co., Ltd. (Jiangsu Province, China). EPSF boxes (outer dimension: 285×165×180 mm, inner dimension: 250×125×145 mm) were purchased from Zhejiang Juewei Technology Co., Ltd. (Zhejiang Province, China). AFP boxes (250×125×145 mm) were purchased from Wenzhou Yahao Packaging Co., Ltd. (Zhejiang Province, China).

Fresh bigeye tuna was removed from the refrigerator at −55°C and packed into three boxes. Samples were randomly divided into three groups. Each box contained 500 g of muscle and 1,000 g of the biological ice bag. Experimental measurements were performed in triplicate for each group. The temperature recorders were set at the center of lids and the lids were sealed with tapes. The entire experiment was conducted in two parts. In the first part, the normal atmospheric temperature process of logistics was simulated and the physicochemical parameters of samples were measured at 0, 8, 16, 24, 32, and 40 h. In the second part, EUL, EPSF, AFP, and fresh bigeye tuna (FT) groups were transported in dry ice from Zhoushan to Shanghai to simulate real logistics.

### Time–temperature monitoring

The temperature-acquisition instrument (Shenzhen Tianpeng Technology Co., Ltd., China) was set at the center of the lid. Time–temperature data were recorded every 15 min during the experiment.

### Physicochemical parameters

#### pH

To assess the pH, 5 g muscle was homogenized with 50 mL distilled water in a beaker at 8,000 r/min. The solution was then maintained at rest for 30 min to obtain a supernatant solution. Finally, the pH value was measured at 20 ± 2°C using a calibrated pH meter (PHSJ-6L, INESA Scientific Instrument Co., Ltd., China).

#### TVB-N

TVB-N content was determined according to the automatic Kjeldahl method as per the GB 5009.228-2016 standard. TVB-N was measured using an automatic Kjeldahl apparatus (JK9870; Focus Technology Co., Ltd., China). Ten grams of samples were homogenized with 75 mL distilled water and soaked for 30 min. TVB-N content was expressed as mg/100 g.

#### Water content and WHC

The water content was determined according to the GB 5009.3-2016 standard. WHC was determined according to the method described by Wang (12) with slight modifications. The filet samples (~3 g), weighed as W_1_, were wrapped with two layers of filter paper and then centrifuged at 5,000 r for 20 min at 4°C. After the residual water on the surface was blotted, samples were weighed again (W_2_). The WHC (%) of the bigeye tuna was calculated according to the following Equation 1:


(1) $$\begin{array}{l} WHC\;\left(\%\right)\;=\;\frac{W_{2}}{W_{1}}\;\times \;100 \end{array}$$

#### Redness

The filet sample was cut into slices with 5 mm thickness. The surface color was measured using a CR-10 colorimeter (Konica Minolta, Japan). Colors were expressed as CIELab coordinates. In this system, a^*^ represents the redness color (13).

#### Metmyoglobin content

The myoglobin content was evaluated according to the method described by Singh (14) with some modifications. Three grams of tuna muscle was homogenized with 30 mL PBS buffer for 30 s at 10,000 rpm; then, the homogenate was centrifuged at 10,000 *g* for 10 min at 4°C. The supernatant was filtered using filter paper; the absorbance was measured at 525, 545, 565, and 572 nm using a UV spectrophotometer (UV-1800; Shimadzu Corp., Japan). The percentage of metmyoglobin was calculated using the following Equation 2:


(2) $$\begin{array}{l} MetMb\;\left(\%\right)\;=\left(-2.514R_{1}+0.777R_{2}+0.800R_{3}+1.098\right)\times \;100 \end{array}$$

where R_1_, R_2_, and R_3_ are the absorbance ratios of A_572_/A_525_, A_565_/A_525_, and A_545_/A_525_, respectively.

### Myofibrillar protein extraction

Myofibrillar protein was extracted according to the method described by Kaewprachu et al. (15) with some modifications. Three grams of bigeye tuna muscle was chopped, 27 ml of 0.05 M KCl was added to it, and then mixed with 0.02 M Tris-Maleic buffer. This mix was homogenized for 1 min, and then centrifuged at 8,000 r/min for 10 min. The supernatant was discarded and the resultant sediments were resuspended again two times under the same conditions. Subsequently, the final precipitate thus obtained was homogenized with 27 ml of 0.6 M KCl-0.02 M Tris-Maleic buffer and extracted at 4°C for 1 h. Finally, the solution was centrifuged at 8,000 r/min for 30 min and the upper solution was obtained separately. The protein content of the myofibrillar proteins was determined using the Biuret method with BSA as a standard.

### Microbiological analysis

The microbial growth was measured according to Hu et al. (16). 25 g of fish muscle was kept in a sterile homogenized bag to which 225 mL 0.85% sterile physiology saline water was added for homogenizing. The tissue homogenate was subsequently diluted 10 times. Three suitable dilutions of the culture were obtained; 1 mL diluent mixed with 15–20 mL Mueller–Hinton agar medium was poured into dishes. After solidification for 1 h, the dishes were turned over and incubated under aerobic and dark conditions at 30°C. The total number of colonies (CFU/g) was determined after 2 days.

### Electronic nose analysis

The flavor characteristics of the tuna slices were analyzed using an E-nose system of PEN3 according to a previously described method (17). The equipment was preheated for ~30 min, and the sampling time was set as 100 s, cleaning time as 100 s, sampling interval as 1 s, zero adjustment time as 10 s, connection sampling time as 10 s, and sample flow as 300 mL/min. Tuna samples (3 g) were placed in a 50 mL beaker and sealed with a preservative film. The samples were maintained at 20°C for ~30 min and then tested on the machine. The experiment was analyzed using the software Win-Muster (Version 1.6.2.22, Airsense Analytics GmbH, Schwerin, Germany). The performance description of the shortcut electronic nose of PEN3 is shown in Table 1.

**Table 1:** Sensors used and their main application in PEN3.

**Order number**	**Sensors**	**Performance description**
S1	W1C	Sensitive to aromatic components such as benzene
S2	W5S	High sensitivity; sensitive to nitrogen oxides
S3	W3C	Ammonia; sensitive to aromatic components
S4	W6S	Mainly selective for hydrogen
S5	W5C	Sensitive to paraffin hydrocarbons aromatic components
S6	W1S	Sensitive to bridle chain paraffin hydrocarbons such as methane
S7	W1W	Sensitive to sulfide
S8	W2S	Sensitive to ethanol
S9	W2W	Aromatic components; sensitive to organic sulfur compounds
S10	W3S	Sensitive to paraffin hydrocarbons

### DNA extraction, PCR amplification, and amplicon sequencing

Genomic DNA of the microbial community was extracted from bigeye tuna tissues using the FastDNA^®^ Spin Kit for Soil (MP Biomedicals, U.S.) according to the manufacturer's instructions. The DNA was analyzed on a 1% agarose gel; the concentration and purity of the DNA were measured using a NanoDrop 2000 UV-vis spectrophotometer (Thermo Scientific, Wilmington, NC, USA). On an ABI GeneAmp^®^ 9700 thermocycler, the hypervariable region V3-V4 of the bacterial 16S rRNA gene was amplified using primer pairs 338F (5′-ACTCCTACGGGAGGCAGCAG-3′) and 806R (5′-GGACTACHVGGGTWTCTAAT-3′; ABI, CA, USA). The 16S rRNA gene was amplified as follows: initial denaturation was conducted at 95°C for 3 min followed by 27 cycles of denaturation at 95°C for 30 s. Annealing was performed at 55°C for 30 s and extension was conducted at 72°C for 45 s followed by a single extension at 72°C for 10 min. The PCR mixtures contained 5× *TransStart* FastPfu buffer (4 μL), 2.5 mM dNTPs (2 μL), five forward primers (5 μM) 0.8 L, five reverse primers (5 μM) 0.8 μL, *TransStart* FastPfu DNA Polymerase (0.4 μL), 10 ng template DNA, and 20 μL ddH_2_O. PCRs were performed in triplicate. The PCR product was extracted from a 2% agarose gel and purified as directed by the manufacturer using AxyPrep DNA Gel Extraction Kit (Axygen Biosciences, Union City, CA, USA) and quantified using QuantusTM Fluorometer (Promega, USA). Purified amplicons were pooled in equimolar and paired-end sequenced on an Illumina MiSeq PE300 platform (Illumina, San Diego, CA, USA) according to the standard protocols provided by Majorbio Bio-Pharm Technology Co. Ltd. (Shanghai, China). The raw reads were deposited into the NCBI Sequence Read Archive (SRA) database under accession number: PRJNA843069.

### Data processing

The raw 16S rRNA gene sequencing reads were demultiplexed, quality-filtered using fastp version 1.2.11 (18), and merged using FLASH version 1.2.7 (19) with the following criteria:

(i) 300 bp reads were truncated at any site receiving an average quality score of <20 over a 50 bp sliding window, and truncated reads shorter than 50 bp were discarded. Reads containing ambiguous characters were also discarded;
(ii) only overlapping sequences longer than 10 bp were assembled according to their overlapped sequence. The maximum mismatch ratio of the overlap region is 0.2. Reads that could not be assembled were discarded;
(iii) samples were distinguished according to the barcode and primers; the sequence direction was adjusted, and the exact barcode was matched. Mismatch of two nucleotides occurred during primer matching. Operational taxonomic units (OTUs) with a 97% similarity cutoff (16) were clustered using UPARSE version 7.1 (20), and chimeric sequences were identified and removed. The taxonomy of each OTU representative sequence was analyzed using RDP Classifier version 2.2 (21) against the 16S rRNA database with a confidence threshold of 0.7. To identify bacterial community richness, community diversity, and sequencing depth, index analyses including number of effective sequence tags, OTUs, Shannon, ACE, Chao 1 Coverage, and coverage were performed using the Usearch (version 11) and Mothur (version 1.30.2). Hierarchical clustering was conducted using R software (version 2.15.3) and QIIME (version 1.9.1).

All values were expressed as mean ± standard deviation of experiments performed in triplicate. The least significant difference was used to test the statistical differences between means among groups and significance was considered at a level of *p* < 0.05. Statistical analysis was conducted using SPSS 22.0 for windows software (SPSS Inc, Chicago, IL, USA). Curves were plotted using Origin Pro 9.1 (OriginLab Cor., Northampton, MA, USA).

## Results and discussion

### Changes in temperature

**Figure 1:**
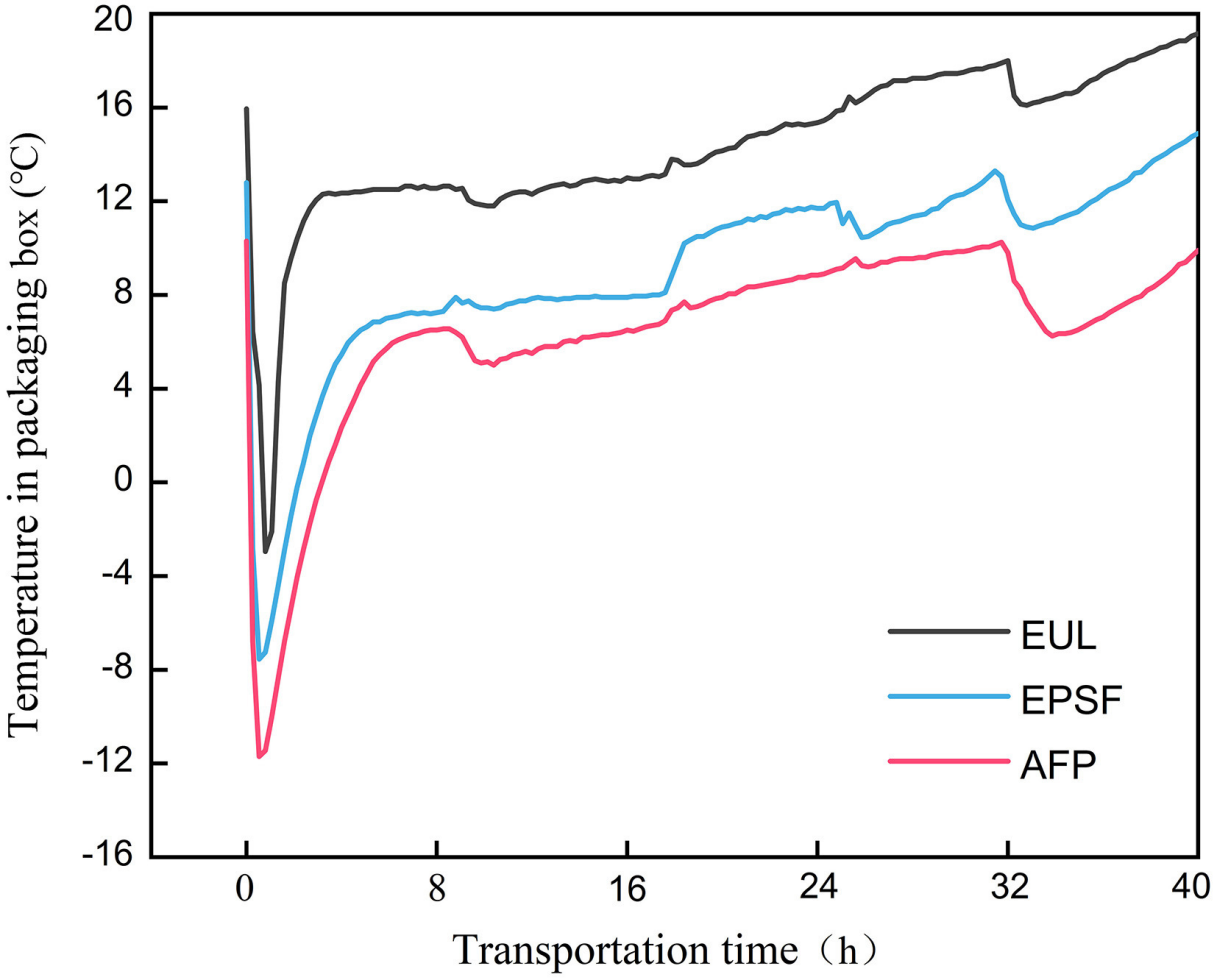
Temperature changes in various logistics packaging boxes during transportation. EUL, European logistics; EPSF, expanded polystyrene foam; AFP, aluminum foil paper.

The temperature change in three types of packages could be divided into three stages as depicted in Figure 1. The first was the rapid cooling stage. Under the combined effect of the ice bag and tuna, the ambient temperature inside quickly dropped below 0°C. The characterization of the second stage was rising fluctuations of temperature. The cooling capacity of the ice bag decreased due to the heat from the external environment. The third was stable stage, wherein, owing to the exhausted cooling capacity, temperature fluctuated with the change of the external environment.

The initial temperature inside the EUL box, EPSF box, and AFP box was 15.95, 12.80, and 10.30°C, respectively. After the first stage, the temperature decreased by 18–22°C and the lowest temperature were −2.95, −7.55, and −11.7°C, respectively. Owing to the melting of the ice bags, the temperature rose gradually, and the temperature of AFP box remained below 8°C. EUL boxes had the highest initial temperature, which implied the worst insulation capacity. Conversely, AFP boxes had the best insulation capacity. The average temperatures during the entire transport process of the three packaging materials were 14.33, 9.14, and 6.35°C. The heating rate of the AFP boxes was the slowest and its final temperature was also considerably lower than that of other groups (Figure 1). Therefore, the AFP box had the best insulation properties because of the high barrier and light shielding properties. Aluminum foil has excellent barrier properties, such as moisture resistance and gas barrier properties, owing to its highly dense metal crystal structure. It can almost completely block the transmission of any gas, water vapor, and light, effectively protecting the contents, preventing the contents from spoilage due to moisture absorption and oxidation, thus significantly extending the shelf life of the package contents (7). The temperature change inside the box was impacted by the periodic change of the external environment. The increase in air temperature was slow at night while the external ambient temperature was low. We observed that the superior insulating capability of aluminum foil material substantially reduced temperature loss and maintained a microenvironment with a lower temperature during transit, thereby reducing tuna quality degradation. The temperature inside the EUL box was high; hence, it is not recommended for use in the logistics process. AFP was the most effective method of preserving a favorable transport microenvironment among the three groups.

### TVB-N and pH analysis

**Figure 2:**
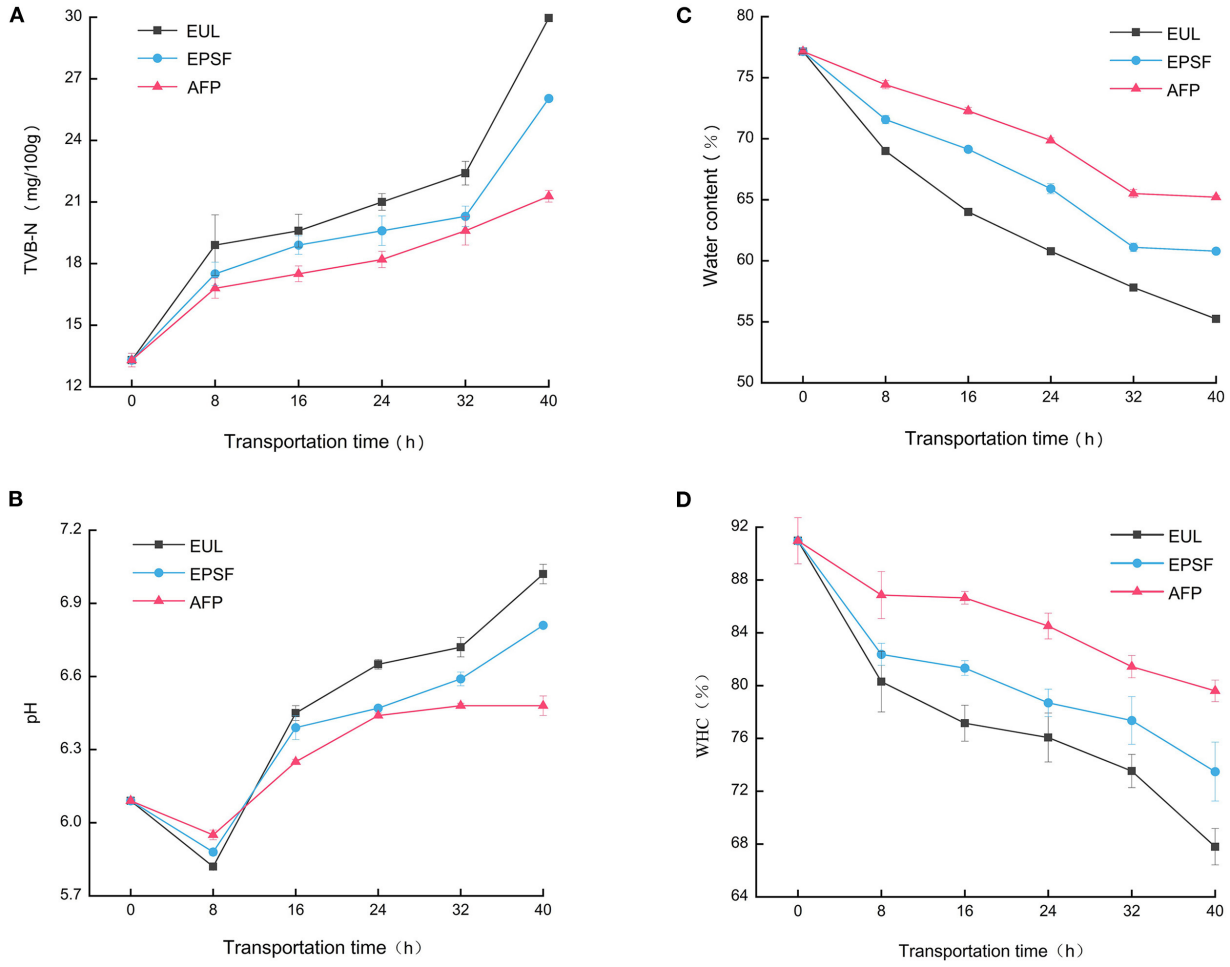
Effects of various logistics packaging boxes on changes in the TVB-N **(A)**, pH **(B)**, water content **(C)**, and WHC **(D)**. EUL, European logistics; EPSF, expanded polystyrene foam; AFP, aluminum foil paper; WHC, water holding capacity. Data were obtained as the mean ± standard deviation (*n* = 3).

Tuna easily degrades into alkaline volatile chemicals, such as ammonia, owing to its high nutritional content. TVB-N is a critical factor to determine the quality of tuna. The higher the content of TVB-N, the faster is the deterioration of the muscle. Generally, when TVB-N value reaches 25 mg/100 g, it is considered inedible (22). TVB-N value of all groups increased during transit, indicating that the quality was rapidly deteriorating (Figure 2A). The initial TVB-N value was 13.30 mg/100 g. After 40 h of transportation, the final value of the three groups increased to 29.96, 26.04, and 21.28 mg/100 g (*p* < 0.05), respectively. While the samples of the EUL and EPSF groups were no longer edible, the sample from the AFP group remained acceptable. The increase in TVB-N may be due to the growth and reproduction of microorganisms at this stage, and then the protein in tuna was decomposed into alkaline nitrogenous substances such as amines (23). For 0–8 and 32–40 h, the increase rate of TVB-N value was faster. This might be due to temperature fluctuations accelerated microorganism propagation and enzymatic reaction, resulting in an increase of ammonia and other nitrogenous substances (12).

Figure 2B shows the pH fluctuations of bigeye tuna in three distinct packaging materials. The initial pH of fresh bigeye tuna samples was 6.09. The pH of tuna after capture was between 6.0 and 6.5. The pH first decreased and then increased with storage time. The same trend was observed by Jinadasa et al. (24). The acidic compounds produced by glycolysis fermentation might have contributed to the reduction in pH value. The increase in pH value may be attributed to the accumulation of nitrogenous substances produced by enzymic oxidation and microbial proliferation (25). After 40 h of transport, the pH of the EUL group showed the highest increase from 6.09 to 7.02, while that of the AFP group showed the lowest increase from 6.09 to 6.48. Generally, the pH value was acceptable up to 6.8; the pH of spoiled fish was above 7.0 (15). The pH value of both the EUL and EPSF groups were above the acceptable range, while that of the AFP group was within the acceptable range. In the environment with temperature fluctuations, the pH value of the EUL group increased significantly (*p* < 0.05) from 6.09 to 7.02 compared to that of fresh tuna, indicating that temperature fluctuations accelerated the quality deterioration of tuna.

### Water retaining capacity analysis

Water content changes dynamically during the storage and transportation stage, which impacts the quality, taste, flavor, and shelf life of tuna. The effect of different transport packages on the water content of bigeye tuna is shown in Figure 2C. The water content of all the three groups decreased in the simulation transportation procedure. This might be due to the denaturation of protein and destruction of muscle tissue owing to the growth and reproduction of microorganisms during transportation, resulting in the large loss of water. The initial value of water content was 77.15%. After 40 h of transportation, the EUL, EPSF, and AFP groups lost 21.92, 16.36, and 11.94%, respectively. The EUL group had the largest decrease, whereas the AFP group had the smallest decrease. This might be because the average temperature in the EUL box was higher, which accelerated the loss of water inside the tuna. However, the aluminum foil material had excellent barrier properties and the average temperature was lower, thus reducing the loss of water.

WHC represents the ability to restrain or add water physically under a certain external force, which is an important indicator that characterizes the degree of muscle tissue damage (26). The WHC decreased with transportation time for different packages (Figure 2D). The WHC of the fresh tuna was 90.97%. The WHC of the three packing conditions decreased with transportation time. The WHC of the EUL group decreased to 67.79% after 40 h, and that of the EPSF and AFP groups decreased to 73.48 and 79.60%, respectively. This could be attributed to the degradation of protein during transportation under the combined action of endogenous enzymes and exogenous microorganisms, making it unable to perform hydration with re-seeded water, resulting in a decrease in WHC. WHC decreased the most at 0–8 h, indicating that the WHC would also be affected by large temperature fluctuation. During the entire process of transportation, the WHC of the AFP group was higher than that of the other two groups, indicating that the barrier of aluminum foil packaging could restrain the loss of water to a certain extent.

### Effects of different transport packaging on the color of bigeye tuna

**Figure 3:**
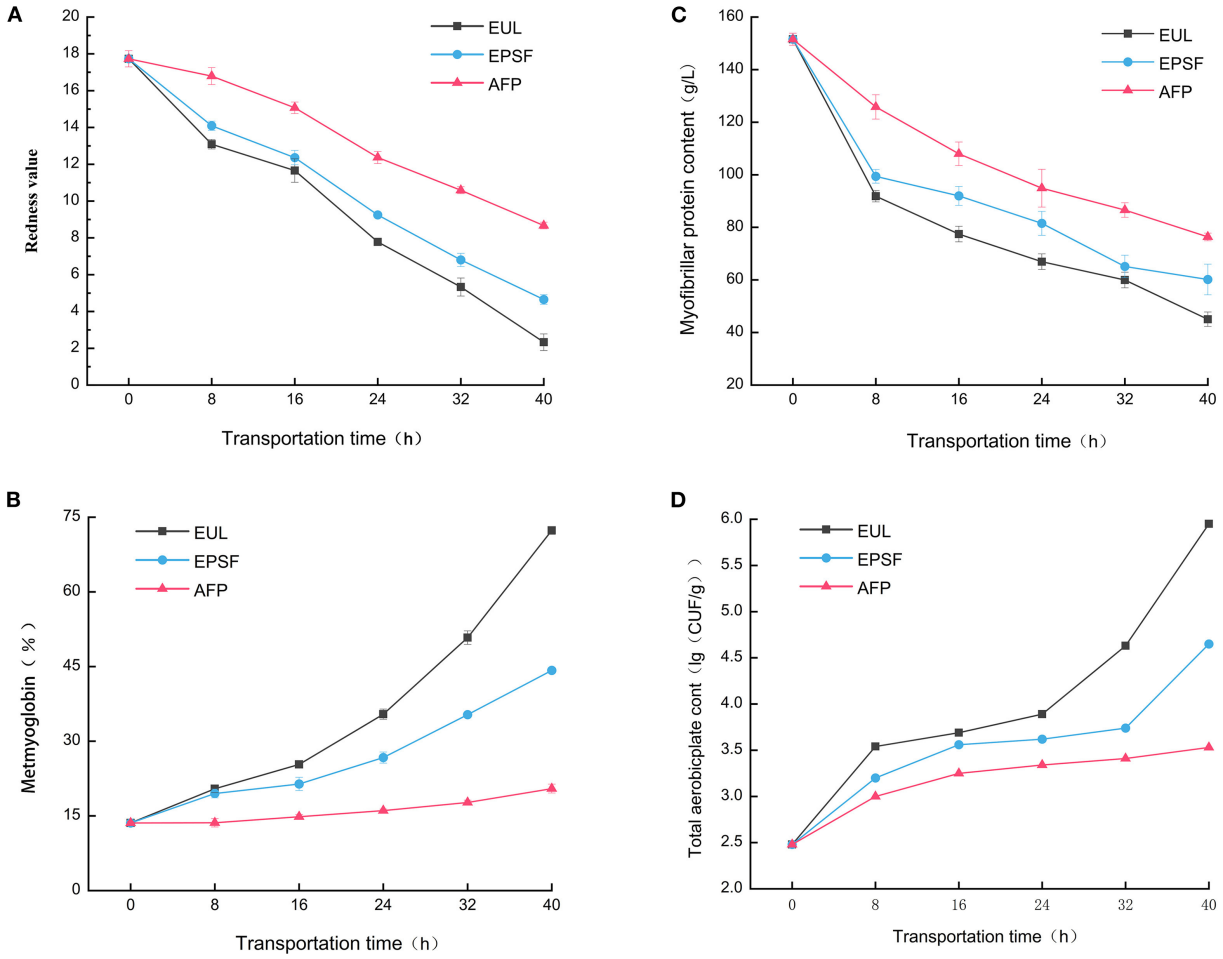
Effects of various logistics packaging boxes on changes in the **(A)** redness value, **(B)** metmyoglobin, **(C)** myofibrillar protein content, and **(D)** total aerobic plate count. EUL, European logistics; EPSF, expanded polystyrene foam; AFP, aluminum foil paper. Data were obtained as the mean ± standard deviation (*n* = 3).

Tuna is relatively expensive and mainly eaten raw. Colors can intuitively reflect the quality of products. Hence, it has become an important factor affecting consumers' willingness to buy the product. Fresh bigeye tuna exhibited a bright red color with a redness rating of 17.73. The redness value of the three groups decreased in varying degrees, and the color of the muscle turned dark as the transportation time increased (Figure 3A). The rate of decline was the fastest at 8 h, possibly because of the temperature change and exposure to oxygen in the air accelerated the oxidation of myoglobin to methemoglobin in fish (27). The color of fish in the EUL group decreased most substantially; the redness value lowered to 2.33 after 40 h, which is significantly (*p* < 0.05) less than the other two groups, indicating severe browning. The muscle color of the fish in the AFP group remained relatively bright red after transportation; the redness value was 8.67, and the color was consistently greater than that of fish in the other two groups. During 0–8 h, the oxidation degree was faster and the decrease rate of the redness value was highest, indicating that severe temperature fluctuations would accelerate the oxidation of fish meat. AFP has the best antioxidant capacity. Dai et al. (28) reported that the oxidation of myoglobin was directly responsible for color deterioration.

Myoglobin is a heme iron-containing protein that acts as an oxygen carrier in muscle cells and is primarily responsible for the color of meat. When the content of metmyoglobin in tuna muscle is <20%, it appears bright red. The higher the content, the darker in color (29). The metmyoglobin concentration of the fresh samples was 13.59%, making the sample appear bright red (Figure 3B). All three groups showed an increasing trend during the transportation process. This was because with the prolongation of circulation time, the oxymyoglobin in the tuna muscle gradually decreased while the metmyoglobin gradually accumulated. The myoglobin was automatically oxidized, which turned the color of the fish meat from the initial bright red to reddish-brown (15). The rise in the redness value also reflected this phenomenon.

After 40 h, the methemoglobin content of both the EUL and EPS groups exceeded 40% and the meat showed browning. For the AFP group, the methemoglobin content was only 20.48%, and the color of the meat was bright red. Pong et al. found that tuna meat stored below 15°C had the least effect on metmyoglobin reduction ability (30). The increase rate of metmyoglobin content of the EUL group increased gradually after 16 h. Combined with the temperature fluctuation curve, the temperature of the EUL group rose to above 15°C after 16 h. Therefore, this indicated that time and fluctuation of temperature affected the color change of tuna. AFP could efficiently maintain the low-temperature environment, preventing the entry of oxygen and inhibiting the oxidation of samples, subsequently maintaining a good color.

### Myofibrillar protein extraction analysis

Myofibrillar protein is the highest protein content in fish; the protein denaturation during storage and transportation of fishes is mainly myofibrillar protein denaturation (31). It is involved in muscle contraction and regulation; in addition, its content and nature directly affect the quality of bigeye tuna. The myofibrillar protein content of all the three groups showed a decreasing trend with an increase in transportation time (Figure 3C). The original concentration of myofibrillar protein in fresh tuna tissue was 105.22 g/L. After 40 h of transportation, the myofibrillar protein content of the EUL logistics box, EPS foam box, and AFP box decreased to 45.05, 60.17, and 76.36 g/L, respectively. The protein content of the EU logistics box decreased the fastest; however, the AFP group had the strongest ability to retain myofibrillar protein. Hu et al. (32) reported that changes in myofibrillar protein content affected the quality of fish, including texture characteristics and WHC. Our findings of WHC analysis were consistent with this report. The decline rate of myofibrillar protein was the largest at 0–8 h and that of the EUL group decreased the most. It was because the myofibrillar protein was a salt-soluble protein, and temperature fluctuations would exacerbate protein denaturation. Owing to the lower ambient temperature in the AFP box, the degree of protein denaturation and enzyme activity was lower. It indicated that aluminum foil packaging could slow down the degradation of myofibrillar protein and has a good effect during transportation and cold preservation.

### Total microbial count of bigeye tuna during transportation

Total plate count is among the important indices to evaluate the freshness of tuna. The colony-forming unit (CFU) of fresh tuna was 2.48 log_10_ CFU/g (Figure 3D). With prolonged transit time and temperature fluctuations, the total plate count of all the three groups showed an increasing tendency. It was mainly because protein autolysis and accumulation of metabolites were favorable to the growth and reproduction of spoilage bacteria. The total plate count of the EPSF and EUL groups was higher than that of the AFP group at the end of the transport, and continued throughout transportation. For 0–8 and 32–40 h, the ascending rate of total plate count of each group was rapid, which was consistent with the TVB-N analytical pattern. Temperature fluctuations accelerated the growth and multiplication of microorganisms. The greater fluctuations and higher degree of temperature, the faster growth and multiplication rate of microorganisms. A longer transit duration at varied transport temperatures led to an increase in the number of colony counts, as reported by Wang et al. (6). After transiting of 40 h, the total plate count of the AFP group was 3.53 log_10_ CFU/g, which remained in an acceptable range, while that of the EUL group reached 5.95 log_10_ CFU/g, which was unsuitable for the standard of raw tuna (33). This demonstrated that aluminum foil packaging materials might significantly reduce temperature fluctuation during the transportation process when compared to the other two packaging materials. As a result, the growth and reproduction of microorganisms were limited, which ensured superior tuna quality.

### Principle component analysis of E-nose

**Figure 4:**
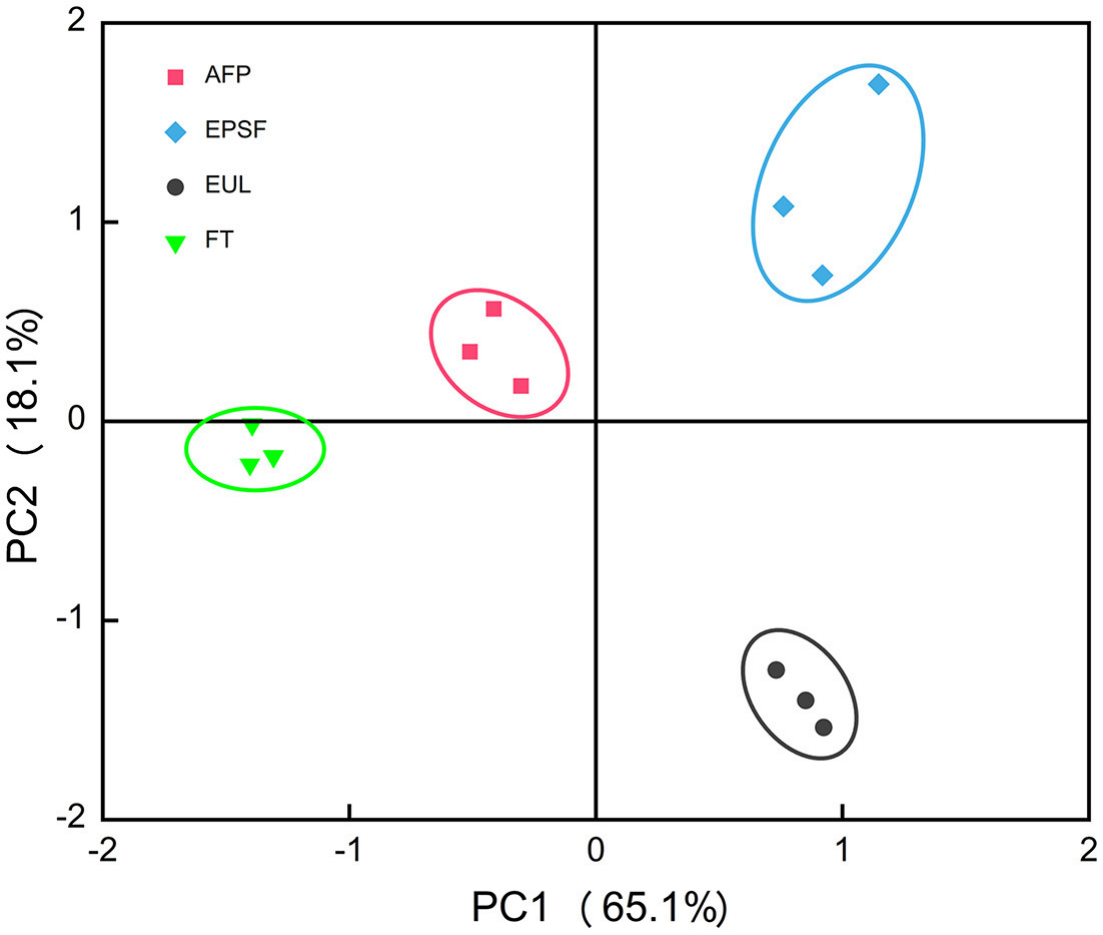
Principal component analysis of the electronic nose data of bigeye tuna transported *via* various logistics packaging boxes. FT, fresh tuna; EUL, European logistics; EPSF, expanded polystyrene foam; AFP, aluminum foil paper.

Flavor is an important index to evaluate the quality of tuna products, which greatly affects consumer purchase. The variance contribution rates of PC1 and PC2 were 65.1 and 18.1%, respectively, and the total variance is 83.2%, indicating that they represented the main characteristic information (17). In principal component analysis (PCA), the relative distance between samples indicated the difference in odor between them. The volatile components of four groups of tuna samples were divided into four regions, indicating that the e-nose could distinguish well between the four groups of samples (Figure 4). All samples of the FT group, EPSF group, and EUL group were in different quadrants, with large distances and separation at the first principal component. This indicated that the samples in these three groups differed significantly from each other, with large sensory differences and considerable flavor differences. This might be owing to the spoilage and deterioration of the samples in generated by different chemicals, resulting in changes of tuna odor. In contrast, the odor of the FT group was closer to that of the AFP group, indicating that there was less variability between the FT and AFP groups, and the odor of the tuna transported in the AFP box was closer to that of fresh samples. This showed that the tuna transported in the AFP box was fresher and maintained flavor better than the other two packaging boxes.

### Microbial community analysis of bigeye tuna during transportation

#### Quality evaluation of 16S rRNA gene sequence of samples

The effective sequencing reads of samples in the FT, AFP, EPSF, and EUL groups were 47,730, 45,159, 32,805, and 45,008, respectively (Table 2). OTUs, Shannon index, ACE index, Chao1 index, and coverage were also examined. All samples had higher than 99% sequencing coverage, implying that the primary microorganisms of bigeye tuna had been detected and the data were sufficient to reflect the microbial diversity of samples. The differences in the number of bacterial OTUs of the three packaging materials and fresh tuna indicated that the three packaging material groups and fresh bigeye tuna had their own balanced microbial community structure and microbial diversity during transportation. The Shannon index, used to measure microbial diversity of the AFP group, was lower than that of the other two groups (Table 2). To measure species richness, the ACE and Chao1 indices were employed. The OTU number, Chao1 index, and ACE index of these samples were in the following order: EUL > EPSF > AFP, indicating that the microbial diversity in the aluminum foil packing group was lower than that in the other groups during the logistics transportation.

**Table 2:** Effects of different packing materials on the diversity of microbial in bigeye tuna.

**Index**	**Packing**	**Transportation time (h)**	
		**0**	**24**
Number of effective sequence tags	AFP box	47,730	45,159
	EPSF box		32,805
	EUL box		45,008
OTUs	AFP box	1,624	413
	EPSF box		659
	EUL box		987
Shannon index	AFP box	4.41	3.77
	EPSF box		4.58
	EUL box		4.29
Ace index	AFP box	488.73	317.24
	EPSF box		429.95
	EUL box		655.11
Chao 1 index	AFP box	495.76	324.87
	EPSF box		437.17
	EUL box		658.94
Coverage	AFP box	99.95	99.97
	EPSF box		99.93
	EUL box		99.92

OTU, operational taxonomic units; FT, fresh tuna; EUL, European logistics; EPSF, expanded polystyrene foam; AFP, aluminum foil paper.

#### Variation in microbial community structures of bigeye tuna under different transportation packaging materials

**Figure 5:**
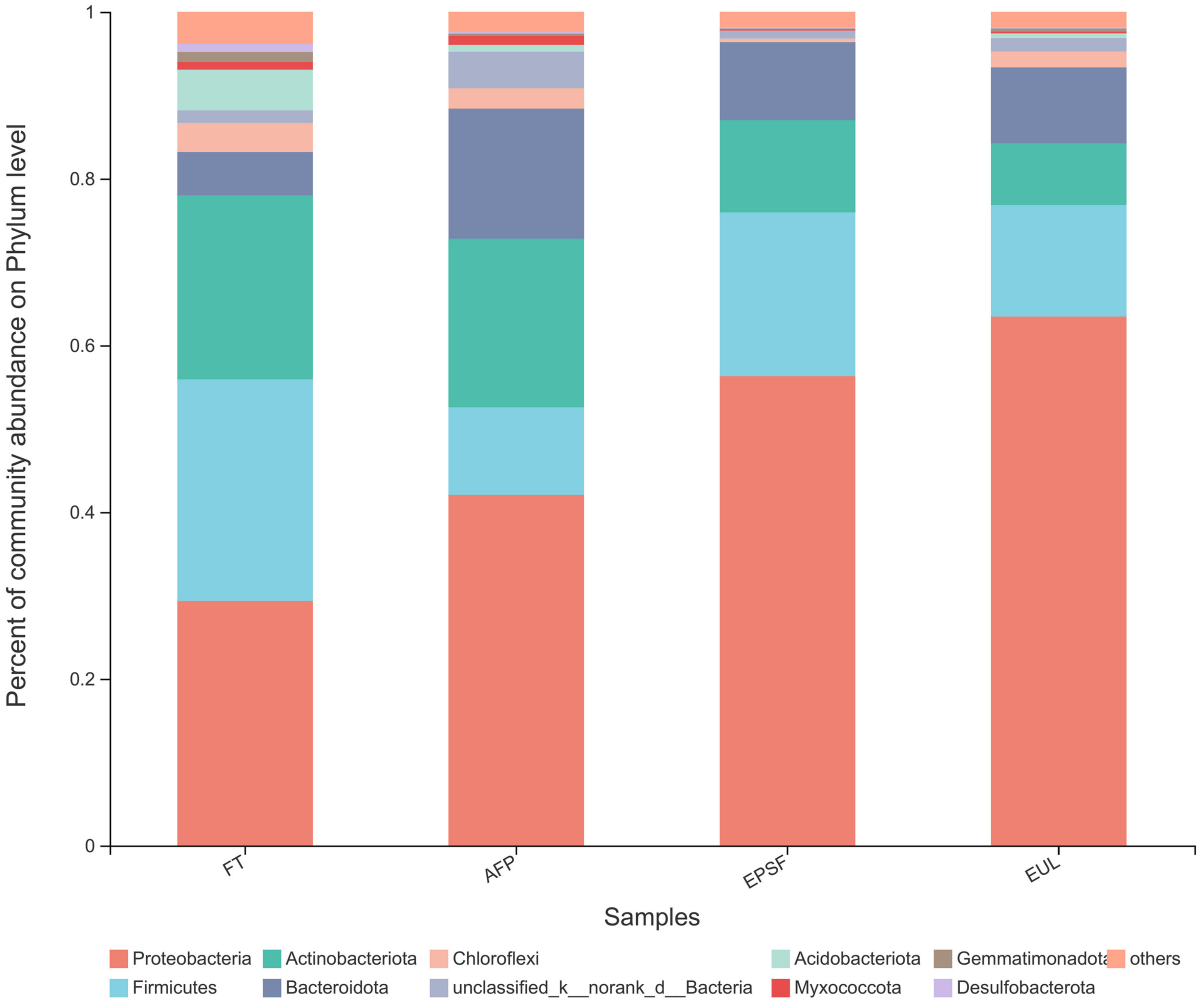
Changes in microbial community structure in bigeye tuna muscle with various logistics packaging boxes at the phylum level. FT, fresh tuna; EUL, European logistics; EPSF, expanded polystyrene foam; AFP, aluminum foil paper.

The species and quantity of bacteria in aquatic products directly affect the quality of the product. The quality of the EUL group deteriorated after transportation, which may have resulted from microorganisms. To reveal the spoilage mechanism of bigeye tuna during transportation, the structural changes and differences of microbial flora during the circulation of bigeye tuna under different packaging methods were investigated. To determine the key microbial members during spoiling, microbial structures were further compared with fresh samples. Ten phyla with relative abundance >1% were detected among the microbial communities of three packaging transportation samples, which included *Proteobacteria, Firmicutes, Actinobacteriota, Bacteroidota*, and *Chloroflexi* (Figure 5). *Proteobacteria* was the most abundant phylum with 29.34% of the sequences in fresh bigeye tuna. The second and third prominent phyla were *Firmicutes* and *Actinobacteriota*, which occupied 26.08 and 22.58%, respectively. Similar to the results of this study, *Proteobacteria* and *Firmicutes* occupied an absolute advantage in meat and meat products in previous studies (34, 35). A similar microbial population was also found in other red flesh fish; for example, in farm-reared Chinook salmon, the majority phylum in freshwater and seawater was also *Proteobacteria*, followed by *Firmicutes* (36).

After 24 h of transportation in the AFP group, *Proteobacteria, Firmicutes*, and *Actinobacteriota* were the top three phyla and occupied 42.05, 10.50, and 20.23%, respectively. In the EUL group, the proportion of *Proteobacteria* increased to 63.44%, followed by *Firmicutes* at 13.40% and *Actinobacteriota* at 7.39%. The highest proportion of *Proteobacteria* in the three transport packages was in the EUL box, followed by the EPSF box and the AFP box (**Figure 7**), suggesting that the microbial structure in AFP was most similar to the fresh sample.

**Figure 6:**
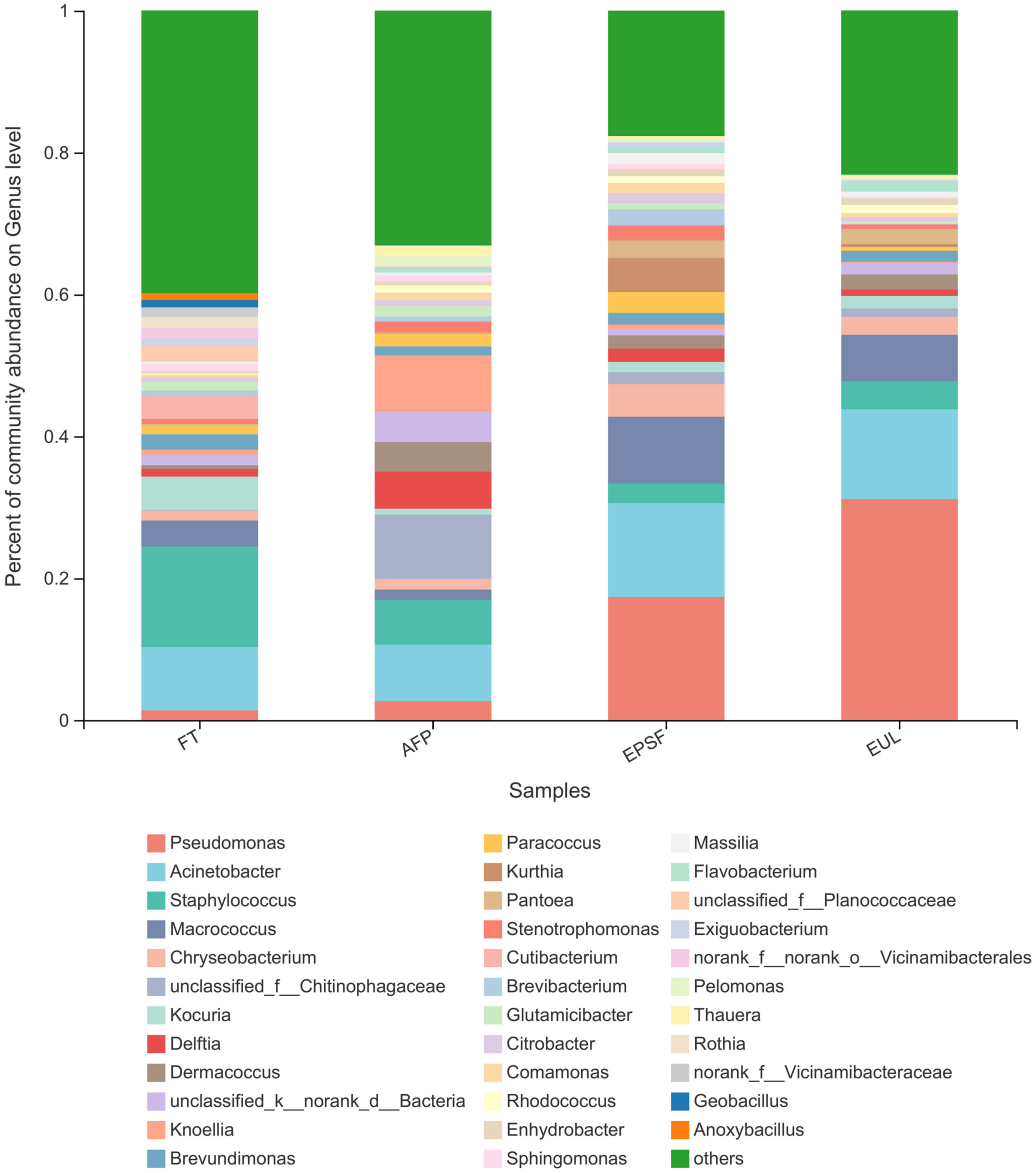
Changes in microbial community structure in bigeye tuna muscle with various logistics packaging boxes at the genus level. FT, fresh tuna; EUL, European logistics; EPSF, expanded polystyrene foam; AFP, aluminum foil paper.

The relative abundances of the major genera varied among three samples (Figure 6). A total of 18 genera were detected in raw samples. The nine major genera, with a relative abundance of >1%, were *Staphylococcus* (14.19%), *Acinetobacter* (8.92%), *Kocuria* (4.73%), *Macrococcus* (3.65%), *Cutibacterium* (3.30%), *Brevundimonas* (2.16%), *Planococcaceae* (2.07%), *Vicinamibacterales* (1.60%), *Rothia* (1.46%), and *Pseudomonas* (1.35%) in the fresh tuna samples. The microbial communities of tuna samples in different transportation packages changed after transportation, indicating different succession patterns. The top three relatively abundant genera in the AFP group were *Chitinophagaceae* (9.03%), *Acinetobacter* (7.97%), and *Knoellia* (7.86%). The relative abundance of *Pseudomonas* in the AFP group samples was 2.68%. The dominant microorganisms in the EPSF and EUL groups were *Pseudomonas, Acinetobacter*, and *Macrococcus*, whose relative abundance values were 17.35, 13.20, and 9.43, 31.16, 12.64, and 6.56%, respectively. The relative abundance of *Pseudomonas* and *Acinetobacter* in both the EUL and EPSF groups was >10%. Combining with the results of TVB-N, total microbial count, and other indicators previously examined, it can be concluded that the samples were already spoiled at the time these measurements were conducted; therefore, *Pseudomonas* and *Acinetobacter* were most likely the main microorganisms responsible for the eventual spoilage of bigeye tuna in these two groups. *Pseudomonas* was the most abundant in the EUL group; its relative abundance was 29.81%, which was higher than that in the FT group. The proportion of *Pseudomonas* was ranked as EUL group > EPSF group > AFP group > FT group.

**Figure 7:**
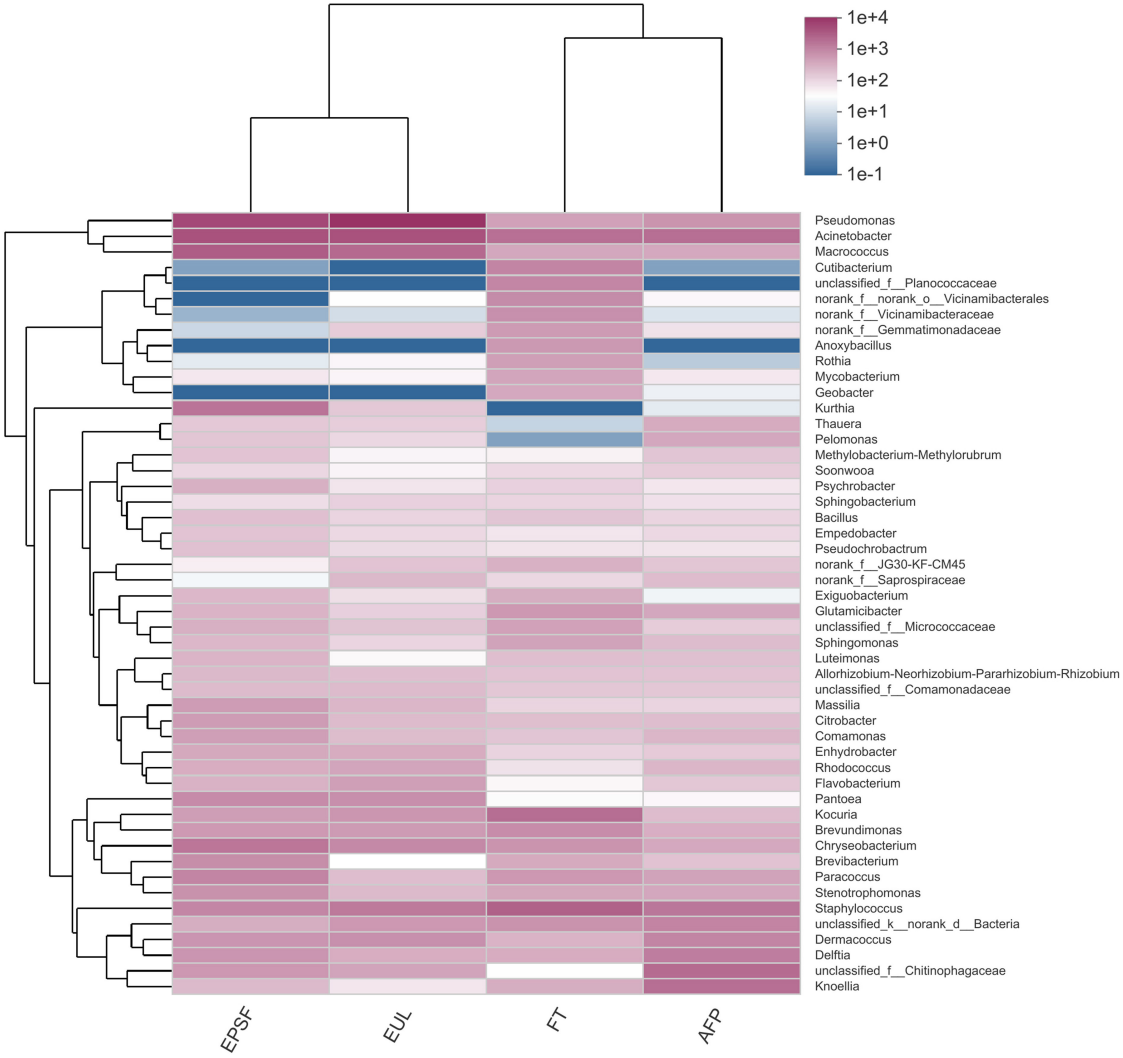
Clustering heat map of microbial abundance at the genus level in bigeye tuna muscle transported by various logistics packaging boxes. FT, fresh tuna; EUL, European logistics; EPSF, expanded polystyrene foam; AFP, aluminum foil paper.

The top 50 abundant genera were selected for heatmap visualization (Figure 7). The EPSF and EUL groups were clustered into one group, and the FT and AFP groups were clustered into another group. Therefore, the FT and AFP groups had higher similarity of the bacterial composition, and the similarity of the EUL group to the EPSF group was higher, indicating that the change in the bacterial community in the AFP group was smaller and closer to the fresh tuna. These results were consistent with the species analysis results. The cluster analysis of bacterial abundance showed that the largest proportion of microbial composition was *Pseudomonas* in both the EPSF and EUL groups. During the transportation of bigeye tuna, the bacterial content of *Pseudomonas, Acinetobacter*, and *Macrococcus* increased substantially and clustered together. The relative content of OTUs corresponding to these three genera was the largest in the OTUs among the entire community. This implied that these genera were the main spoilage bacteria, and the number of bacterial communities increased gradually during the transportation, thus becoming the dominant bacteria during the transportation of these two groups of bigeye tuna. The relative abundances of *Pseudomonas, Acinetobacter*, and *Macrococcus* in the FT and AFP groups were lower than those in the first two groups, which suggesting that AFP box packaging can reduce the growth and reproduction of spoilage microorganisms.

*Pseudomonas* was the dominant spoilage bacteria of fresh meat during the storage terminal stage (37). The proteolysis ability of *Pseudomonas* enabled them to seep into the meat and use nutrients to occupy the niche conducive for their growth, which was most susceptible in the later storage periods (38). When compared with the fresh samples, the relative abundance of *Pseudomonas* in the AFP group increased by 1.34%, and the relative abundance of *Staphylococcus* and *Acinetobacter* decreased by 0.95 and 7.93%, respectively. The relative abundance of *Pseudomonas* in the AFP group was significantly lower than that in the EUL and EPSF groups, which meant that the growth of *Pseudomonas* was inhibited.

In summary, the bacterial community structure of the AFP group, which was distinct from that of the EUL and EPSF groups, was relatively similar to that of the FT group. This indicated that AFP materials could effectively reduce the variation in microbial structures during tuna transportation. The low air permeability of AFP materials might ensure the stability of the internal environment during transportation and inhibit the growth of aerobic spoilage dominant bacteria.

## Conclusion

In this study, the effects of three transport packaging (EUL box, EPSF box, and AFP box) on the logistics quality and microbial diversity of bigeye tuna were studied. We found that the AFP box could inhibit the growth of potential spoilage microbes, reduce the rise of TVB-N, redness value, metmyoglobin content, keep better color, and flavor of bigeye tuna. The results of microbial diversity indicated that the microbial diversity of bigeye tuna in the EUL group and EPSF group significantly changed, compared with that of fresh tuna. The microbial colonies in the EUL and EPSF boxes were similar, while the microbial community of the AFP box was closer to that of the FT group. The dominant phyla in all samples were *Proteobacteria, Firmicutes*, and *Actinobacteriota*. At the genus level, *Pseudomonas, Acinetobacter*, and *Macrococcus* were the dominant bacteria in the EUL and EPSF groups, but the proportion of *Pseudomonas* in the EPSF group was higher than that in the EUL group; the dominant bacteria in the AFP group were *Chitinophagaceae, Acinetobacter*, and *Knoellia*, which was similar to that of the FT group (the dominant bacteria: *Staphylococcus, Acinetobacter*, and *Kocuria*). Aluminum foil has excellent barrier properties on water, oxygen, light, and microorganisms, which are the main factors causing food spoilage. AFP boxes can effectively inhibit the diversity of microbial flora and the growth of microorganisms, guaranteeing the quality of bigeye tuna preferably during transportation.

The bigeye tuna stored in the AFP box was slowly thawed in the process of logistics, and the temperature rise rate was lower than that of the EUL box and EPSF box. The quality of bigeye tuna can be guaranteed during the logistics. These findings implied that the AFP box had the best insulation properties. AFP box can maintain the temperature inside the box for 5 h, and can be applied to the short-distance distribution of fresh aquatic products, which is more economical and convenient energy-saving. EUL boxes were not recommended for transporting perishable products as the bigeye tuna had been deteriorated by the end of logistics. AFP packaging not only light-shielding, but also exhibited heat retention, fragrance preservation, non-toxicity, and odorlessness. Considering the importance of food safety, the results of our study could provide valuable data on the logistics transportation of bigeye tuna, and may also be of practical use in overcoming the challenges of managing the cold chain during storage and transportation. However, the temperature during actual transportation is often more diverse, changes of bigeye tuna quality and microorganisms under different transport temperatures are also quite different, and further research is needed to understand the effects of various material logistics boxes on bigeye tuna under different transportation temperatures.

## Data availability statement

The datasets presented in this study can be found in online repositories. The names of the repository/repositories and accession number(s) can be found at: https://www.ncbi.nlm.nih.gov/, PRJNA843069.

## Author contributions

ZC and WP contributed to the investigation, methodology, formal analysis, data curation procedures, and writing the original manuscript draft. WX contributed to validation of methodology and reviewing and editing the draft. JY and XW contributed to validation, investigation, resources, and supervision. SB, AG, and SD contributed to validation and reviewing and editing the draft. XY contributed to conceptualization, project administration, supervision, and reviewing and editing the draft. All authors have read and agreed to the published version of the manuscript.

## Funding

This work was financed by the Scientific Research Business of Colleges and Universities in Zhejiang Province (Project No. 2021JZ010), National Key R&D Projects (Project No. 2020YFD0900901), National Key Research and Development Program of China (Project No. 2018YFC1602101), National Natural Science Fund of China (Project No. 32001622), National Natural Science Foundation of China (Project No. 32072291), Zhejiang Leading Training Program (2021R51006), Project of Zhoushan (2022C31060), and was supported by Zhejiang Provincial Sannongjiufang Program (2022SNJF069).

## Conflict of interest

The authors declare that the research was conducted in the absence of any commercial or financial relationships that could be construed as a potential conflict of interest.

## Publisher's note

All claims expressed in this article are solely those of the authors and do not necessarily represent those of their affiliated organizations, or those of the publisher, the editors and the reviewers. Any product that may be evaluated in this article, or claim that may be made by its manufacturer, is not guaranteed or endorsed by the publisher.

## Abbreviations

FAO: Food and Agriculture Organization of the United Nations
DHA: Docosahexaenoic acid
EPA: Eicosapentaenoic acid
HDE: high-density polyethylene
EUL: European logistics
EPSF: Expanded polystyrene foam
AFP: Aluminum foil paper
FT: Fresh tuna
WHC: Water holding capacity
PCA: Principal component analysis
OUT: Operational taxonomic units.

## References

[1] Yi ZK Xie J. Assessment of spoilage potential and amino acids deamination and decarboxylation activities of *Shewanella putrefaciens* in bigeye tuna (*Thunnus obesus*). LWT Food Sci Technol. (2022) 156:113016. 10.1016/j.lwt.2021.113016

[2] FAO. Fishery and Aquaculture Statistics 2019. FAO Yearbook. Rome: FAO (2021). p. 12.

[3] Ndraha N Hsiao HI Vlajic J Yang MF Li HTV. Time-temperature abuse in the food cold chain: review of issues, challenges, and recommendations. Food Control. (2018) 89:12–21. 10.1016/j.foodcont.2018.01.027

[4] Bai B Zhao K Li X. Application research of nano-storage materials in cold chain logistics of e-commerce fresh agricultural products. Result Phys. (2019) 13:10249. 10.1016/j.rinp.2019.01.083

[5] Tapilatu Y Nugraheni PS Ginzel T Latumahina M Limmon GV Budhijanto W. Nano-chitosan utilization for fresh yellowfin tuna preservation. Aquatic Procedia. (2016) 7:285–95. 10.1016/j.aqpro.2016.07.040

[6] Wang XY Xie J Qian YF. A non-invasive method for quantitative monitoring of quality changes and water migration in bigeye tuna (*Thunnus obesus*) during simulated cold chain logistics using low-field nuclear magnetic resonance coupled with PCA. Food Sci Technol Int. (2020) 26:475–84. 10.1177/1082013220903148 32070144

[7] Afrianto E Rochima E Liviawaty E Marsvia SA. Shelf life of nori from *Eucheuma cottonii* with aluminum foil packaging based on the accelerated shelf life test method. Asian J Fish Aquatic Res. (2020) 10:7–15. 10.9734/ajfar/2020/v10i330182

[8] Yang X Zhu L Zhang Y Liang R Luo X. Microbial community dynamics analysis by high-throughput sequencing in chilled beef longissimus steaks packaged under modified atmospheres. Meat Sci. (2018) 141:94–102. 10.1016/j.meatsci.2018.03.010 29606393

[9] Yu D Zhao W Yang F Jiang Q Xu Y Xia W. Strategy of ultrasound-assisted processing to improve the performance of bio-based coating preservation for refrigerated carp fillets (*Ctenopharyngodon idellus*). Food Chem. (2021) 345:128862. 10.1016/j.foodchem.2020.128862 33338838

[10] Ekonomou SI Parlapani FF Kyritsi M Hadjichristodoulou C Boziaris IS. Preservation status and microbial communities of vacuum-packed hot smoked rainbow trout fillets. Food Microbiol. (2022) 103:103959. 10.1016/j.fm.2021.103959 35082076

[11] Jaaskelainen E Jakobsen LMA Hultman J Eggers N Bertram HC Bjorkroth J. Metabolomics and bacterial diversity of packaged yellowfin tuna (*Thunnus albacares*) and salmon (*Salmo salar*) show fish species-specific spoilage development during chilled storage. Int J Food Microbiol. (2019) 293:44–52. 10.1016/j.ijfoodmicro.2018.12.021 30639999

[12] Wang JF Yu WH Xie J. Effect of glazing with different materials on the quality of tuna during frozen storage. Foods. (2020) 9:231–47. 10.1145/3412815 32098131 PMC7074287

[13] Fazial FF Ling TL Ahmad AAA Zubairi SI. Physicochemical changes of tuna fish (*Euthynnus affinis*) throughout refrigerated storage condition. A preliminary study. AIP Conf Proc. (2019) 2111:050002. 10.1063/1.5111250

[14] Singh A Benjakul S Zhang B Deng S Mittal A. Effect of squid pen chitooligosaccharide in conjugation with different modified atmospheric packaging conditions on color and storage stability of tuna slices. Food Control. (2021) 125:108013. 10.1016/j.foodcont.2021.108013

[15] Kaewprachu P Osako K Benjakul S Suthiluk P Rawdkuen S. Shelf life extension for Bluefin tuna slices (*Thunnus thynnus*) wrapped with myofibrillar protein film incorporated with catechin-Kradon extract. Food Control. (2017) 79:333–43. 10.1016/j.foodcont.2017.04.014

[16] Hu JJ Xu YJ Majura JJ Qiu YH Ding JJ Hatab S. Combined effect of the essential oil and collagen film on the quality of pacific mackerel (*Pneumatophorus japonicus*) fillet during cold storage. Foodborne Pathog Dis. (2021) 18:455–61. 10.1089/fpd.2021.0007 34096803

[17] Pan WC Benjakul S Sanmartin C Guidi A Ying XG Ma LK. Characterization of the flavor profile of bigeye tuna slices treated by cold plasma using E-Nose and GC-IMS. Fishes. (2022) 7:13–27. 10.3390/fishes7010013

[18] Chen S Zhou Y Chen Y Gu J. Fastp: an ultra-fast all-in-one FASTQ preprocessor. Bioinformatics. (2018) 34:884–90. 10.1093/bioinformatics/bty560 30423086 PMC6129281

[19] Magoc T Salzberg SL. FLASH: fast length adjustment of short reads to improve genome assemblies. Bioinformatics. (2011) 27:2957–63. 10.1093/bioinformatics/btr507 21903629 PMC3198573

[20] Edgar RC. UPARSE highly accurate OTU sequences from microbial amplicon reads. Nat Methods. (2013) 10:996–8. 10.1038/nmeth.2604 23955772

[21] Wang Q Garrity GM Tiedje JM Cole JR. Naive bayesian classifier for rapid assignment of rRNA sequences into the new bacterial taxonomy. Appl Environ Microbiol. (2007) 73:5261–7. 10.1128/AEM.00062-07 17586664 PMC1950982

[22] Wang XY Xie J. Comparison of physicochemical changes and water migration of *acinetobacter johnsonii, Shewanella putrefaciens*, and cocultures from spoiled bigeye tuna (*Thunnus obesus*) during cold storage. Front Microbiol. (2021) 12:727333. 10.3389/fmicb.2021.727333 34777276 PMC8586447

[23] Shi R Li Y Liu L. Synergistic anti-oxidative and antimicrobial effects of oat phenolic compounds and ascorbate palmitoyl on fish balls during cold storage. J Food Sci. (2021) 86:4628–36. 10.1111/1750-3841.15922 34549438

[24] Jinadasa BKKK Galhena CK Liyanage NPP Yildiz F. Histamine formation and the freshness of yellowfin tuna (*Thunnus albacares*) stored at different temperatures. Cogent Food Agr. (2015) 1:1028735. 10.1080/23311932.2015.1028735

[25] Sun L Sun J Liu D Fu M Yang X Guo Y. The preservative effects of chitosan film incorporated with thinned young apple polyphenols on the quality of grass carp (*Ctenopharyngodon idellus*) fillets during cold storage: correlation between the preservative effects and the active properties of the film. Food Packaging Shelf. (2018) 17:1–10. 10.1016/j.fpsl.2018.04.006

[26] Cropotova J Mozuraityte R Standal IB Ojha S Rustad T Tiwari B. Influence of high-pressure processing on quality attributes of haddock and mackerel minces during frozen storage, and fishcakes prepared thereof. Innov Food Sci Emerg. (2020) 59:102236. 10.1016/j.ifset.2019.102236

[27] Singh A Benjakul S Zhou P Zhang B Deng S. Effect of squid pen chitooligosaccharide and epigallocatechin gallate on discoloration and shelf-life of yellowfin tuna slices during refrigerated storage. Food Chem. (2021) 351:129296. 10.1016/j.foodchem.2021.129296 33640769

[28] Dai Y Lu Y Wu W Lu XM Han ZP Liu Y. Changes in oxidation, color and texture deteriorations during refrigerated storage of ohmically and water bath-cooked pork meat. Innov Food Sci Emerg. (2014) 26:341–6. 10.1016/j.ifset.2014.06.009

[29] Chow CJ Ochiai Y Hashimoto K. Effect of freezing and thawing on autoxidation of bluefin tuna myoglobin. Nippon Suisan Gakkaishi. (1985) 5:2073–8. 10.2331/suisan.51.2073

[30] Pong CY Chiou TK Ho ML Jiang ST. Effect of polyethylene package on the metmyoglobin reductase activity and color of tuna muscle during low temperature storage. Fisheries Sci. (2000) 66:384–9. 10.1046/j.1444-2906.2000.00059.x

[31] Liu X Sun X Wei Y Ma Y Sun P Li X. Effects of ultrasonic treatment on physico-chemical properties and structure of tuna (*Thunnus tonggol*) myofibrillar proteins. J Food Compos Anal. (2022) 108:104438. 10.1016/j.jfca.2022.104438

[32] Hu YM Zhang NH Wang H Yang YF Tu ZC. Effects of pre-freezing methods and storage temperatures on the qualities of crucian carp (*Carassius auratus* var. pengze) during frozen storage. J Food Process Pres. (2020) 45:15139. 10.1111/jfpp.15139

[33] Nelce Mailoa M Marthina Tapotubun A Matrutty TEAA. Analysis total plate counte (TPC) on fresh steak tuna applications edible coating caulerpasp during stored at chilling temperature. IOP Conf Ser Earth Environ Sci. (2017) 89:012014. 10.1088/1755-1315/89/1/012014

[34] Li D Zhang J Song S Feng L Luo Y. Influence of heat processing on the volatile organic compounds and microbial diversity of salted and vacuum-packaged silver carp (*Hypophthalmichthys molitrix*) fillets during storage. Food Microbiol. (2018) 72:73–81. 10.1016/j.fm.2017.11.009 29407407

[35] Li X Li C Ye H Wang Z Wu X Han Y. Changes in the microbial communities in vacuum-packaged smoked bacon during storage. Food Microbiol. (2019) 77:26–37. 10.1016/j.fm.2018.08.007 30297053

[36] Steiner K Heasman K Laroche O Pochon X Preece M Bowman JP. The microbiome of Chinook salmon (*Oncorhynchus tshawytscha*) in a recirculation aquaculture system. Aquaculture. (2021) 534:736227. 10.1016/j.aquaculture.2020.736227

[37] Pellissery AJ Vinayamohan PG Amalaradjou MAR Venkitanarayanan K. Spoilage bacteria and meat quality. Meat Qual Anal. (2020) 17:307–34. 10.1016/B978-0-12-819233-7.00017-3

[38] Nychas GJE Skandamis PN Tassou CC Koutsoumanis KP. Meat spoilage during distribution. Meat Sci. (2008) 78:77–89. 10.1016/j.meatsci.2007.06.020 22062098

